# Repair calvarial defect of osteoporotic rats by berberine functionalized porous calcium phosphate scaffold

**DOI:** 10.1093/rb/rbab022

**Published:** 2021-06-01

**Authors:** Dahao Wang, Peng Zhang, Xifan Mei, Zhenhua Chen

**Affiliations:** 1 Liaoning University of Traditional Chinese Medicine, Shenyang 110847, China; 2 Jinzhou Medical University, Jinzhou 121001, China

**Keywords:** berberine, porous ceramics, BMSCs, osteoporosis

## Abstract

In this article, we propose a simple scheme of using berberine (BBR) to modify porous calcium phosphate ceramics (named PCPC). These BBR molecules regulate the crystallization of hydroxyapatite nanorods on PCPC. We found that these nanorods and the adsorbed BBR changed the interface micro-environment of PCPC by SEM images. The microenvironment of PCPC surface is essential for promoting BMSCs’ proliferation and differentiation. These results demonstrated that PCPC/BBR markedly improved the bone regeneration of osteoporosis rats. Moreover, PCPC/BBR had significantly increased the expression levels of ALP, osteocalcin and bone morphogenetic protein2 and RUNX2 in BMSCs originated from osteoporosis rats.

## Introduction

Osteoporosis is a common systemic metabolic bone disease, which usually causes bone loss, increased bone fragility and bone microstructure damage [1]. With the growth of age, the body will have different degrees of osteoporosis symptoms, such as bone pain, low back pain, fracture, hunchback and so on. According to statistics, in the osteoporosis population, the incidence rate of female is far greater than that of male. This is due to the decrease of estrogen secretion and ovarian function in postmenopausal women, which is called postmenopausal osteoporosis [2]. The main manifestations of osteoporosis are loss of bone mass and increase of bone fragility [3]. At the same time, osteoporosis patients will also have changes in bone morphology, structural disorders between trabeculae and trabeculae, low back and hip pain, persistent muscle pain in the body, the probability of fracture in patients with late greatly increased [4]. The purpose of the treatment of osteoporosis is to prevent or delay bone loss, avoiding fracture risk, or even increasing the bone mass of patients.

In the past 30 years, with the development of tissue engineering, great progress has been made in the development of seed cells, scaffold materials and growth factors. Among them, ceramic scaffold material is an important carrier, which has great significance for the treatment of osteoporosis [5, 6]. Among many ceramic materials, calcium phosphate cements (CPCs) are one of the most representative biomaterials [7–12]. *Construction of biomimetic natural wood hierarchical porous structures bioceramic with micro/nano whisker coating to modulate cellular behavior and osteoinductive activity.* CPCs are a kind of inorganic materials with various calcium phosphate salts as the main component, which has osteogenic activity, degradation activity and self-curing ability under physiological conditions, and are suggested to have high stability and osteogenic potential compared with autologous bone grafts [13–15]. Among them, the most noteworthy is porous CPC (PCPC), which can promote the growth of cells into its pores, and then repair the defects. In addition, abundant calcium and phosphorus ions at PCPCs surface can induce the proliferation and differentiation of BMSCs by regulating bone formation and repair proteins, containing osteopontin, osteocalcin (OCN) and bone morphogenetic protein-2 (BMP2) [16]. Many reports have pointed out that porous calcium phosphate can promote mesenchymal stem cells because it can firmly adsorb related proteins [17–19], such as BMP2, platelet-derived growth factor, OCN and so on. It has been reported that the osteoinductive ability of PCPCs might be related to activation of BMP/Smad and Wnt/β-Catenin pathway [20, 21], which may be related to porosity and the surface roughness of calcium phosphate [22–27].

Berberine (BBR) is the main antibacterial component of Coptis chinensis. It has been used in the treatment of intestinal infection for hundreds of years [28]. BBR has many biochemical functions, such as anti-inflammatory, immune regulation, hypoglycemic, anticancer and so on. At the same time, our previous studies also showed that BBR has a strong ability to promote recovery [29]. Therefore, we proposed BBR functionalized porous calcium phosphate ceramics (PCPC/BBR) repaired bone defect.

## Experimental

All the experiments were carried out according to the requirements of the research ethics committee of Jinzhou Medical University.

### Preparation of samples

PCPC were prepared by the reported method [19, 30], purchased from Biomaterials Research Center, Sichuan University. PCPC/BBR was produced as followings. At first, PCPC plate was soaked into 15 ml PBS solution (0.01 mol/l) in a conic flask. Subsequently, 10 μM BBR was dissolved in the above solution (Supplementary Fig. S1). The flask was put still for 24 h at 4°C. Then, the PCPC plate and the solution were put into a 50 ml autoclave and reacted at 110°C, for 10 h. At last, brown color PCPC/BBR disk was obtained.

### Animal experiments

Thirty female *Sprague–Dawley* rats (SD rats) (3 months old) were divided into three groups (*n* = 10/group). The specific procedures were as follows: all SD rats were anesthetized with 2% Pentobarbital Sodium (0.3 ml/100g), and then bilateral oophorectomy was performed [31]. Estradiol levels were too low to be detected in Ovx animals, at the assayed time points. It proves the success of osteoporosis model from the side. Then Ovx animals were divided into three groups. (i) Control (calvarian defect only), (ii) PCPC (calvarian defect implanted with PCPC) and (iii) PCPC/BBR (calvarian defect implanted with PCPC/BBR).

### Preparation of BMSCs

Bone marrow was collected from the femora of osteoporosis rats [32]. The collected cells were placed on a 60-mm plate and added with low glucose DMEM (low glucose DMEM was added with 10% fetal bovine serum (10%), 100 IU/ml penicillin and 100 μg/ml streptomycin). Low glucose DMEM was purchased from Invitrogen company. The well was then ensconced in a 37°C, 5% CO_2_ incubator for 48 h. After 48 h, the medium was changed. When the cells are about 80–90% of the culture dish, they can be subcultured. After two passages, the next experiment can be carried out.

### BMSCs culture in PCPC, PCPC/BBR and normal medium

BMSCs’ proliferation in PCPC, PCPC/BBR and normal medium was determined by MTT method. First, the third generation BMSCs were digested and centrifuged, and then cultivated into 96-well plates with 5000 cells per well for 72 h. Then, the extracts of PCPC and PCPC/BBR collected in advance were replaced (the control group was treated with normal medium). Each piece of PCPC and PCPC/BBR was immersed in 1 ml medium and collected 24 h later. After 6, 12 and 24 h of culture with the extract and normal medium respectively, the medium was aspirated and 20 μl MTT (5 mg/ml, in PBS solution) was added. After incubated for 3–4 h, MTT solution was pipetted out and 150 μl DMSO was put on into each well. After 15 min, quantitative analysis was carried out by a microplate reader at 490 nm.

### ALP level assay

BMSCs cultured for 14 days were fixed in 10% neutral formalin for 15 min. Removed the fixed solution and clean it with PBS for three times. Then we added the prepared ALP staining kit (Beyotime, China) to the dish, covered the sample and stained at 37°C for 30 min. The dye was then removed and washed three times with PBS, which washed for 5 min each time. Finally, it was observed under the microscope.

### Alizarin red S staining

The third generation BMSCs was randomly divided into con-group, PCPC group and PCPC/BBR group with three dishes in each group. After 24 h of normal culture, the medium of each group was changed into the extract. Cont Group continued to use normal medium. The medium and extract were altered every 3 days. After 14 days, the cells were fixed in 10% neutral formalin for 15 min. Then we removed the fixed solution and clean it with PBS for three times. Next, we added alizarin red S staining solution (Beyotime, China) to the dish, covered the sample and stained for 5 min. Then, the liquid in the well was removed and washed three times with PBS. Finally, it was observed under the microscope. Calcium deposition positive cells were orange red.

### Western blot

At 14 days treatment, the cells were lysed in Pipa buffer and collected. Next, the protein was added into SDS-PAGE sample loading buffer for standby. Then isolated the protein on 10% SDS-PAGE and transferred it to the membrane by semi dry transfer device. Then the PVDF membrane was sealed with 5% milk for 2 h, and then incubated with primary antibody at 4°C overnight. The first antibody was prepared with Antibody Penetration Buffer (Invitrogen, America). After rinsing with the washing solution, we incubated the immune complexes with horseradish peroxidase labeled anti rabbit or anti-mouse IgG and incubated the PVDF membrane at room temperature for 2 h. Finally, we detected using the ECL Plus Western Blotting Detection Kit (Beyotime, China). All the primary antibodies used were purchased from the Abcam company. The specific information is as follows: anti-BMP2 (ab214821), anti-ALP (ab214821), anti-OCN (ab133612), anti-RUNX2 (ab76956) anti-beta-actin (ab8227).

### Surgical craniotomies

SD rats were anesthetized with 2% Pentobarbital Sodium (0.3 ml/100 g, intraperitoneal injection). A 1.5-cm sagittal incision was made on the scalp to expose the skull through blunt separation. In the exposed calvarian field of vision, two circular calvarian defects with a critical size of 5 mm in diameter were developed with a dental trephine, and then PCPC and PCPC/BBR were implanted, respectively. Three experiment groups were: control group: only calvarian defect without material implantation. PCPC group: PCPC was implanted in calvarian defect. PCPC/BBR group: PCPC/BBR was implanted in skull defect. At 8 weeks after operation, the rats were killed, and their calvarian defect were fixed in 4% paraformaldehyde solution and placed at 4°C for further analysis.

### Micro-CT analysis

The rats were killed 8 weeks after the operation, and the skulls were excised, trimmed and fixed in 4% paraformaldehyde solution for 2 days. The samples were scanned by micro-CT and reconstructed by ctvox software (skyscan, Bruker). Then, bone mineral density (BMD) and bone volume/total volume (BV/TV) fraction were measured by software.

### Histological analysis

Each sample was dehydrated by alcohol gradient (70–100%), and then implanted in polymethylmethacrylate. After the samples were observed to be hardened, the longitudinal section of the skull was cut into 100-μm thick slices with a slicer (SLEE, Germany). Afterwards, the slices were dyed with van Gieson’s to estimate bone tissue. Bone is represented by red areas and the black area represents PCPC or PCPC/BBR. ImageJ (America) was used to quantitatively evaluate the area of new bone formation on four randomly selected sections.

### Statistical analysis

Spss19.0 statistical software was used for one-way analysis of variance. All results were expressed in the form of mean ± SD (*x* ± SD). *P* < 0.05 indicated that the difference was statistically significant.

## Results

### Characterization of PCPC and PCPC/BBR

**Figure 1.**
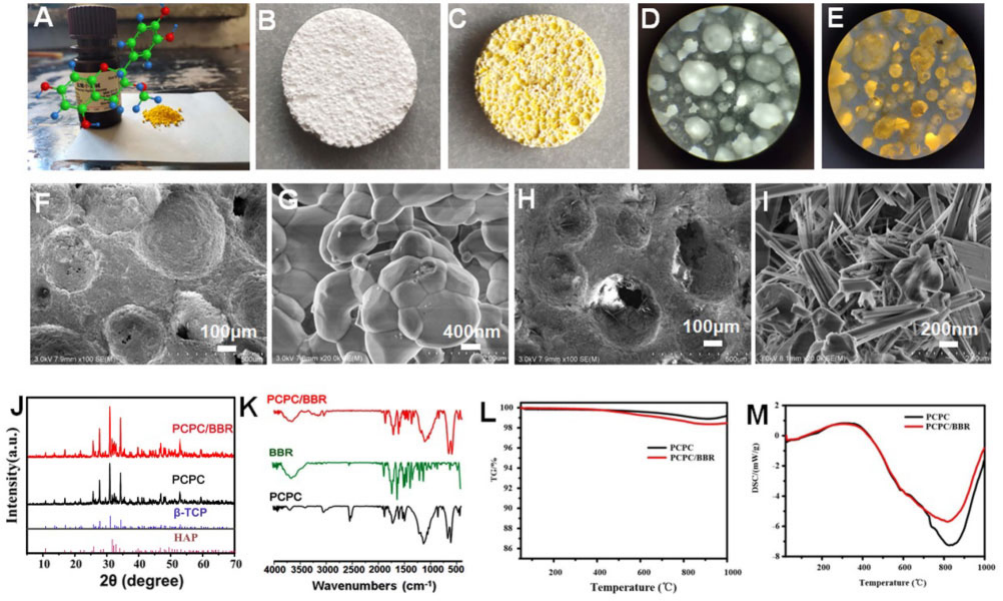
(**A**) Photo of BBR powder and its structure; (**B**) photo of PCPC scaffold; (**C**) photo of BBR functionalized porous calcium phosphate (PCPC/BBR) ceramic scaffold; stereo microscope image of (**D**) PCPC, and PCPC/BBR (**E**); SEM images of (**F, G**) PCPC and (**H, I**) PCPC/BBR; (**J**) XRD reflections of PCPC and PCPC/BBR; (**K**) FTIR spectra of PCPC, BBR and PCPC/BBR; (**L**) TGA curves PCPC and PCPC/BBR; (**M**) DSC curves PCPC and PCPC/BBR.

Figure 1 provides the micro surface structure and other basic characterization of the samples. Figure 1A provided the molecular structure and the color appearance information of BBR. Figure 1B (PCPC) and C (PCPC/BBR) indicates that both PCPC and PCPC/BBR were distributed with pores (diameter ranged from 0.1 μm to 2 mm). Furthermore, stereo microscope images of (Fig. 1D) PCPC and (Fig. 1E) PCPC/BBR showed that PCPC and PCPC/BBR both have good porous light transmittance under light irradiation. However, Fig. 1F–I showed the surface morphology of PCPC and PCPC/BBR were different. PCPC’s rough surface was composed by micro crystal (grain size of 1–5 μm, Fig. 1F and G). Although PCPC/BBR’s surface was occupied by nanorods (Fig. 1H). Figure 1I further provided the detail information of those nanorods were 1–2-μm long and 1 0–30 nm in diameter, and these nanorods were also assembled into nanobeams with the thickness of 1 0 0–300 nm. In the PBS solution, under the acidic microenvironment of BBR, under hydrothermal conditions, the surface of the ceramic sheet was remineralized to produce the nanorods as the SEM image in Fig. 1I illustrated. In order to study the surface morphology, porosity and the pore size distribution of the samples, the physical adsorption tests and Brunner–Emmet–Teller measurements were carried out. The results showed that the specific surface area of PCPC was lower than that of PCPC/BBR, while the pore size of PCPC/BBR was smaller than that of PCPC (Supplementary Fig. S2). The XRD pattern in Fig. 1J shows that the XRD pattern of PCPC/BBR with nanorods on the surface was not significantly different from that of PCPC. When compared with standard cards, the material used was biphasic calcium phosphate, which was made up of beta-tricalcium phosphate (β-TCP) and hydroxylapatite (HAP; standard spectral cards for β-TCP and HAP were PDF#09-0169 and PDF#72-1243, respectively) [33]. These suggested that the nanorods spread all over the surface of PCPC/BBR were well-crystallized hydroxyapatite.

Figure 1 shows BBR play an indispensable role in changing PCPC’s morphology. This may be due to the interactions between BBR and PCPC, like hydrogen bonding between BBR and PCPC’s phosphate group, and the electrostatic interactions between BBR and PCPC. The FT-IR absorptions at 1616, 1365, 1185 and 1112 cm^−1^ (ascribed to BBR, Fig. 1K) might indicate that BBR has been incorporated into PCPC/BBR. PCPC/BBR promoted BMSCs proliferation, differentiation and bone formation. Thermogravimetric analysis (TGA; Fig. 1L) and differential scanning calorimetry (DSC; Fig. 1M) proved that PCPC and PCPC/BBR had thermal weight loss at 4 0 0–800°C. When compared with PCPC, PCPC/BBR lost more weight. The difference in thermal weight loss between PCPC and PCPC/BBR at 4 0 0–800°C was 0.6 wt%. This result suggested that the content of BBR compounded into PCPC/BBR was 0.6 wt%. Release dynamics of BBR from PCPC/BBR monitored by UV-vis experiments *in vitro*. The results present in Supplementary Fig. S3 proved that PCPC served a carrier for sustained release of BBR as long as 9–10 days.

### PCPC/BBR promotes proliferation and differentiation of BMSCs

**Figure 2.**
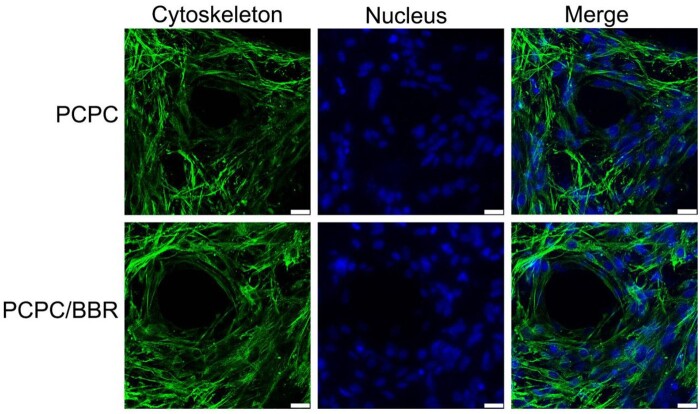
CLSM images of BMSCs cultured with PCPC and PCPC/BBR for 1 day (scale bar: 25 µm).

**Figure 3.**
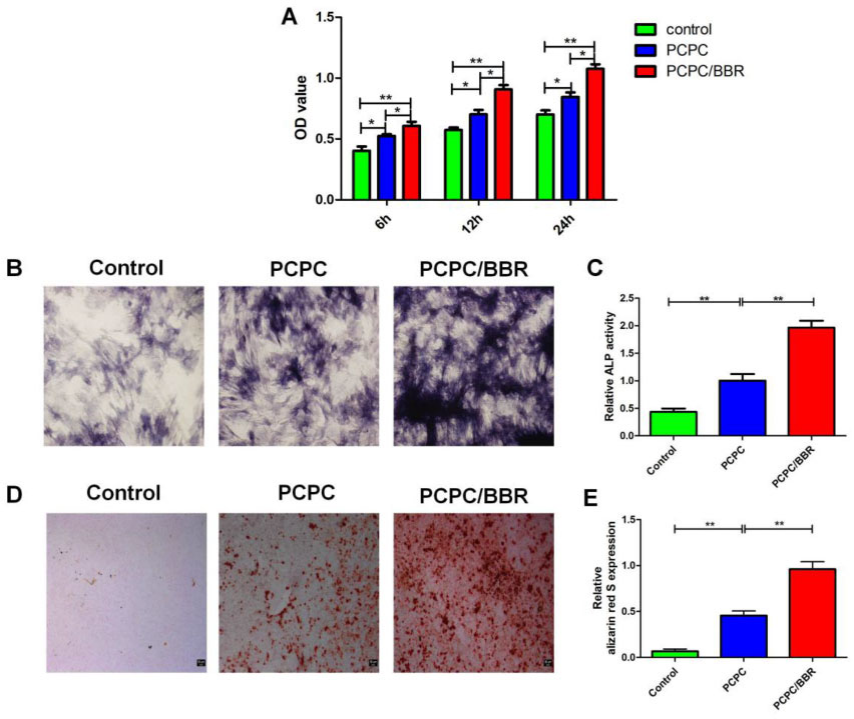
(**A**) The effect of PCPC and PCPC/BBR on the proliferation of BMSCs was evaluated by the MTT. (**B**) ALP staining, (**C**) quantitative analysis of ALP, (**D**) alizarin red S staining and (**E**) alizarin red S activity were used to evaluate the effect of PCPC, PCPC/BBR on osteogenic differentiation of BMSCs (*P < 0.05; **P < 0.01; scale bar: 50 µm).

**Figure 4.**
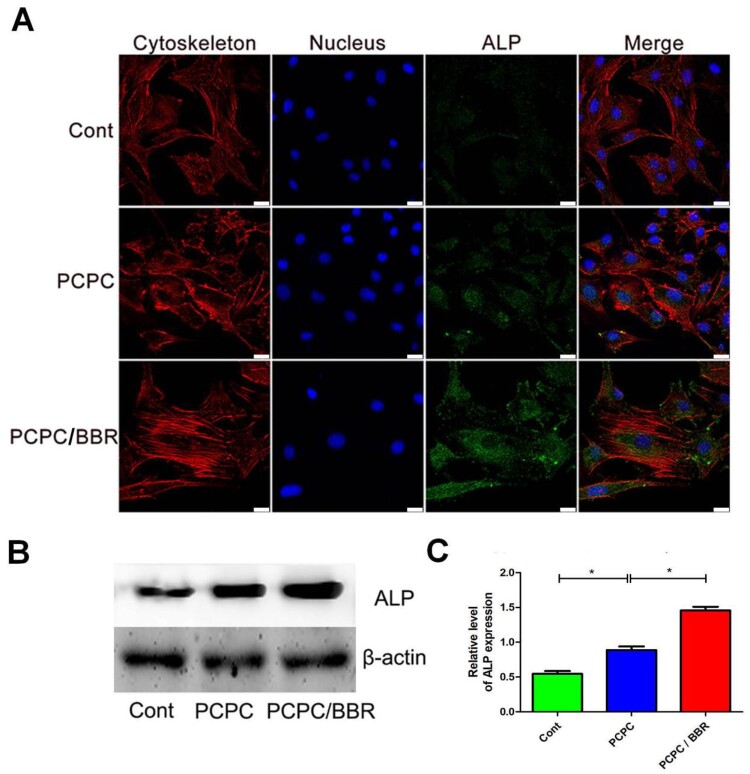
CLSM images of the level of ALP. In the image, the PCPC group was cultured with the extract of the PCPC medium, the PCPC/BBR was cultured with the extract of the PCPC/BBR medium, and the control group was cultured in the normal medium. The expression of ALP was detected by WB (**B**) and quantitative analysis (**C**). Actin was used as control (*P < 0.05; scale bar: 25 µm).

**Figure 5.**
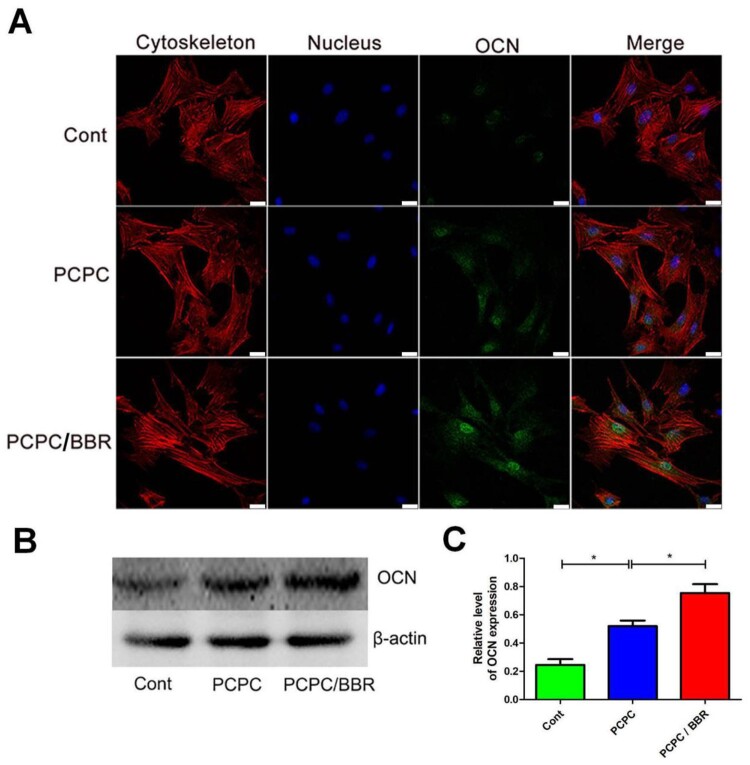
CLSM images of the level of OCN. In the image, the PCPC group was cultured with the extract of the PCPC medium, the PCPC/BBR was cultured with the extract of the PCPC/BBR medium, and the control group was cultured in the normal medium. The expression of OCN was detected by WB (**B**). β-actin was used as control (*P < 0.05; scale bar: 25 µm).

**Figure 6.**
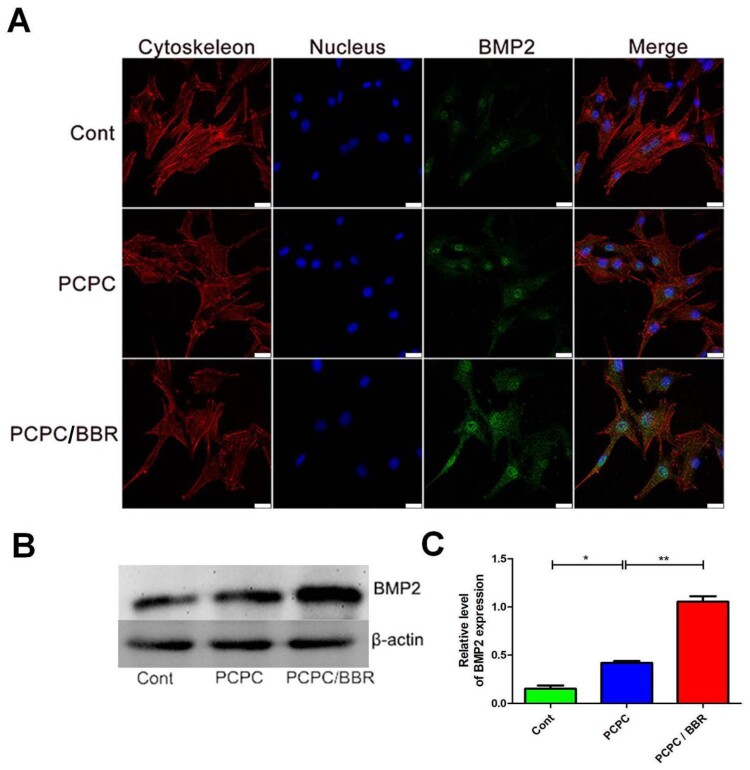
CLSM images of the level of BMP2. In the image, the PCPC group was cultured with the extract of the PCPC medium, the PCPC/BBR was cultured with the extract of the PCPC/BBR medium, and the control group was cultured in the normal medium. The expression of BMP2 was detected by WB (**B**). β-actin was used as control (*P < 0.05; **P < 0.01; scale bar: 25 µm).

**Figure 7.**
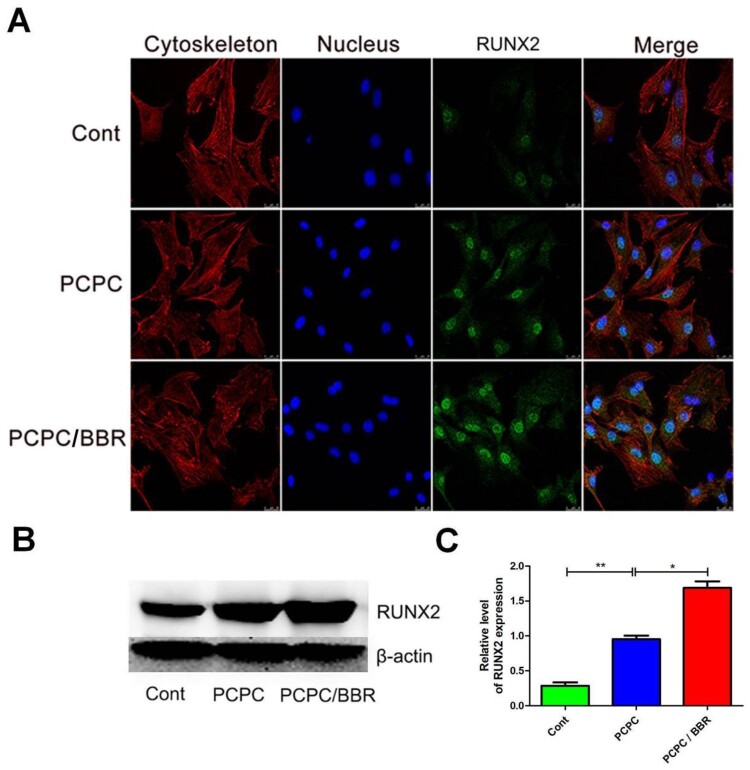
CLSM images of the level of RUNX2. In the image, the PCPC group was cultured with the extract of the PCPC medium, the PCPC/BBR was cultured with the extract of the PCPC/BBR medium, and the control group was cultured in the normal medium. The expression of RUNX2 was detected by WB (**B**). β-actin was used as control.(*P < 0.05;**P < 0.01; scale bar: 25 µm).

First, the BMSCs of the third generation are co-cultured with different concentrations of BBR for one day. It is found that the activity of BMSCs is the strongest at the concentration of 10 μM (Supplementary Fig. S1). Figure 2 shows the effects of PCPC and PCPC/BBR on the growth of BMSCs. Corresponding to the results of cell activity, PCPC/BBR has stronger adhesion to BMSCs, and cells can grow better in pores, expand more and have better cell activity. Through co-culture *in vitro*, we can see that PCPC/BBR could effectively accelerate the proliferation of BMSCs. Figure 3A presents the detailed information of mesenchyma stem cells cultured in PCPC, PCPC/BBR and the blank control groups. We can easily see that PCPC/BBR can significantly promote BMSCs’ proliferation. At the same time, we can also see that with the enhanced of culture time, the proliferation trend of PCPC/BBR is more obvious. In Fig. 3B, the expression of ALP protein in PCPC/BBR is markedly higher than that in the other two groups. In addition, the quantitative analysis of ALP activity (Fig. 3C) shows that the PCPC/BBR group is significantly different from the control group and PCPC group (*P* < 0.05). Figure 3D shows a significant increase in mineral deposition in the PCPC/BBR group compared with the control group. At the same time, the activity of alizarin red S is also quantitatively analyzed (Fig. 3E). After statistics, compare with the control group and PCPC group, PCPC/BBR group at 14 days, the difference is marked (*P* < 0.05; Figs 4–7).

### Micro-CT analysis

**Figure 8.**
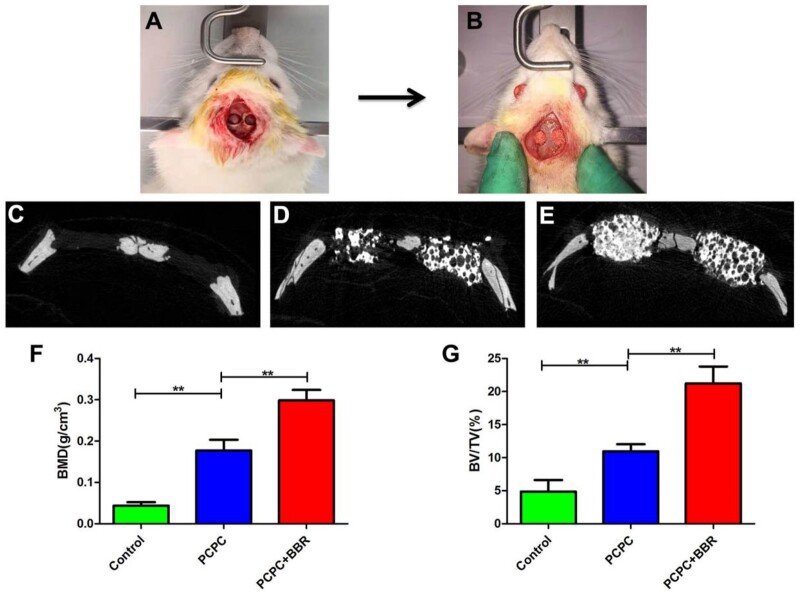
(**A, B**) Picture of calvarial defect in rats. (**C–E**) Micro-CT reconstruction images of con-group, PCPC group and PCPC/BBR group. (**F**) Micro-CT BMD of each group. (**G**) Morphometric analysis of BV/TV; (* indicates significant differences, **P < 0.01).

**Figure 9.**
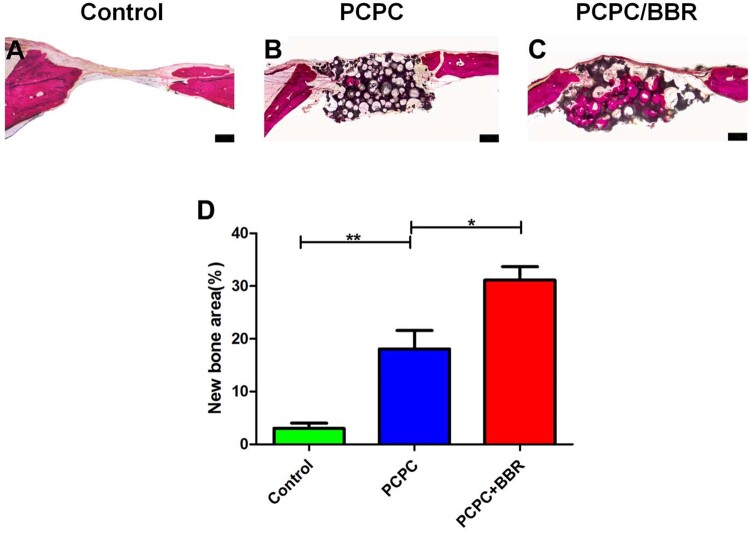
Histological results of PCPC/BBR promoted bone regeneration. (**A–C**) The undecalcified skulls were seliced and dyed with van Gieson’s. The new formed bone and PCPC residue were illustrated in red and black color, respectively. (**D**) Eight weeks after implantation, the percentage of new bone area was assessed by histomorphometric analysis (*P < 0.05; **P < 0.01; scale bar: 1 mm).

The calvarial defect of rats is shown in Fig. 8A and B. Figure 8C–E shows 3D micro-CT reconstruction images. Sagittal observation exhibits that the number of new bone formation in PCPC/BBR group is significantly more than that in PCPC group and control group. The new bone is quantitatively analyzed by the analysis system. Analysis shows that the local BMD of PCPC/BBR group is markedly higher than that of PCPC group or control group (*P* < 0.05; Fig. 8F). In addition, BV/TV value analysis show the similar difference with BMD data (Fig. 8G). These results proved that PCPC/BBR could promote bone regeneration in osteoporosis.

van Gieson staining results of non-calcified specimens exhibit that new bone is significantly raised in PCPC/BBR group. New bone formation area is markedly higher than those of PCPC group and control group (Fig. 9A–C). There is also marked difference between PCPC group and Control group (Fig. 9D). The results of van Gieson staining are consistent with those of micro-CT.

## Discussion

As aging is becoming more and more serious, more and more elderly people are suffering from osteoporosis [34]. Obviously, osteoporosis has threatened the health of the elderly [35, 36]. There are more and more patients with osteoporosis, and they tend to be younger. Sex hormones, decreased calcium absorption, inflammatory factors and other factors may cause osteoporosis [37]. Bone matrix is the component of bone, which is composed of organic matter and inorganic salt. The organic matter mainly refers to the mucopolysaccharide outside the glial fibers. Most of the inorganic substances are precipitated in the glial fibers and arranged in parallel along the long axis of the fiber, which has strong anti-pressure function, mainly including calcium phosphate, calcium carbonate and so on [38, 39]. Inorganic salts are distributed in the organic matter of bone in the form of hydroxyapatite crystals, and the amorphous calcium phosphate develops into hydroxyapatite crystals, which are buried in the gap of the organic matter of bone, commonly known as bone mineralization [40].

At present, due to the existence of calcium phosphate and hydroxyapatite in human bones, calcium phosphate-based ceramics have become suitable biomaterials. It can induce bone formation, promote the osteogenic differentiation of bone marrow mesenchymal stem cells, and regulate the crystallization of minerals [9], but the differentiation promoting ability of calcium phosphate ceramics needs to be strengthened. Currently, the drugs to prevent osteoporosis, especially traditional Chinese medicine, mainly promote the proliferation and differentiation of osteoblast-related cells. BBR can promote the differentiation of osteoblasts [41]. BBR was modified on PCPC to prepare PCPC/BBR. The levels of RUNX2, BMP2, OCN and ALP were measured *in vitro* to prove that PCPC/BBR can promote the proliferation and differentiation of mesenchymal stem cells. *In vivo* experiments show that PCPC can further promote the osteoporotic rat skull defect. Promoting the expression of these proteins is of great significance for osteoporosis and improvement of BMD in aged mice.

## Conclusion

In short, in this study, BBR was modified on PCPC to synthesize PCPC/BBR. Cell experiments first proved the biocompatibility of PCPC/BBR and the osteogenic differentiation of BMSCs. Animal experiments show that PCPC/BBR can significantly increase the bone repair and promote the BMD in osteoporotic rats. Meanwhile, PCPC/BBR could promote the expression of RUNX2, BMP2, OCN and ALP in BMSCs. In the future, the molecular mechanism of PCPC/BBR promoting bone repair needs further study.

## Supplementary data

Supplementary data are available at *REGBIO* online.

## Supplementary Material

rbab022_Supplementary_Data
