# Supplementary material for: Repair calvarial defect of osteoporotic rats by berberine functionalized porous calcium phosphate scaffold
Source: Regen Biomater. 2021 Jun 1;8(3):rbab022. doi: 10.1093/rb/rbab022 (PMC8240619; doi:10.1093/rb/rbab022)
Supplement: rbab022_Supplementary_Data [file rbab022_supplementary_data.zip › supporting information.doc]

**Supporting Information**

Figure S1 The effect of different concentrations of berberine on the proliferation of BMSCs was evaluated by the MTT.

Figure S2 N_2_ adsorption/desorption isotherms and pore size distribution (inset) of the PCPC (A) and PCPC/BBR (B).

Figure S3 (A) Uv-vis absorption of BBR in PCPC/BBR at different time and (B) in vitro release profile of BBR from PCPC/BBR.
