# Supplementary material for: Repair calvarial defect of osteoporotic rats by berberine functionalized porous calcium phosphate scaffold
Source: Regen Biomater. 2021 Jun 1;8(3):rbab022. doi: 10.1093/rb/rbab022 (PMC8240619; doi:10.1093/rb/rbab022)
Supplement: rbab022_Supplementary_Data [file rbab022_supplementary_data.zip › Response.docx]

**Response to the Editorial corrections and reviewers’ comments**

Dear Editor,

We are very grateful for the reviewers’ valuable comments which have greatly contributed to this revised manuscript. When submitting this revised manuscript together with the annotated version, we also present our answers to the reviewers’ comments meticulously one by one in the following pages.

We are expecting that this revised manuscript and our answers to the reviewers’ comments would be helpful for your consideration of publishing this study in your esteemed journal.

Your assistance to this submission would be greatly appreciated.

Best wishes,

Sincerely,

Zhenhua Chen

PhD, Professor

Jinzhou Medical University, Jinzhou, PR China

**Reviewer 1**

***Q1******:*** *It would be better to* *quantify the surface morphology of different scaffolds.*

**Answer:** We greatly appreciate the reviewer’s precious comment. The surface morphology of different scaffolds was quantified as followings: we conducted the physical adsorption tests to study the surface morphology. The results showed that the surface area of PCPC was 0.5871 m²/g and the surface area of 1.7089 m²/g was achieved for PCPC/BBR (Figure S2).

Figure S2 N_2_ adsorption/desorption isotherms and pore size distribution (inset) of the PCPC (A) and PCPC/BBR (B).

***Q2:*** *While the work reports CaP ceramic scaffolds, some recent works on the CaP ceramic-polymer composites on the bone regeneration should be cited and discussed also as below (some examples are below):*

*- Zhang D et al., The development of collagen based composite scaffolds for bone regeneration, Bioactive Mater, 2018, 3, 129.*

*- Ziquan Wu et al. Biomimetic and osteogenic 3D silk fibroin composite scaffolds with nano MgO and mineralized hydroxyapatite for bone regeneration. J Tissue Eng, 2020; 11: 2041731420967791.*

**Answer:** We greatly appreciate the reviewer’s precious comment. The suggested excellent papers about bone regeneration have been cited.

***Q3:*** *Authors need to rationale why the markers for analyses were selected.*

**Answer:** We greatly appreciate the reviewer’s precious comments. At that time, the laboratory only had ACtin protein, so I could only choose it. Although the molecular weight of the bone-related protein to be detected is very close to that of actin, we first develop the actin, then take out the membrane for rinsing, and then incubate the primary antibody to develop the target protein.

***Q4:*** *It would be better to quantify the bone quality as well.*

**Answer:** We greatly appreciate the reviewer’s precious comments. We usually use bone mineral density（BMD）to represent bone mass, and BMD indicators have been measured in each group in Figure 8F.

**Reviewer 2**

***Q1***: *SEM images show that the adsorbed BBR changes the surface morphology of PCPC scaffolds. Please determine porosity of the scaffolds before and after adsorption of BBR.*

**Answer:** Thanks for the reviewer’s precious comment. We obtained the porosity of PCPC was 0.1164% and the porosity of PCPC/BBR was 0.6137%.

***Q2:*** *Please indicate the characteristic peaks of BBR in XRD and FTIR spectra.*

**Answer:** We greatly appreciate the reviewer’s precious comments. We explored the crystal structure of PCPC/BBR through XRD. In Figure 1J, by comparing the XRD diffraction peaks of PCPC and PCPC/BBR, we did not see the obvious diffraction peaks of BBR. Therefore, BBR didn’t change the crystal form of PCPC, and PCPC still maintained a complete structure. In Figure 1K, the characteristic peaks of BBR in FTIR spectra were as follows: 1616 cm^-1^ (benzene, C=C), 1506 cm^-1^ (benzene, C=C), 1365 cm^-1^ and 1277 cm-1 (=C-O-), 1230 cm^-1^ (=C-O-), 1034 cm^-1^ (C-O-). These were the infrared characteristic peaks of BBR. The characteristic peaks of PCPC/BBR in FTIR spectra, we could also see the existence of characteristic peaks of benzene ring and =C-O-, which proved the successful connection of BBR and PCPC.

***Q3:*** *BBR seems to play a role in promoting proliferation and differentiation of BMSCs. So, the authors need to determine the release profile of BBR from the scaffolds.*

**Answer:** We greatly appreciate the reviewer’s precious comment. The release profile of BBR from the scaffolds was as followings:

Figure S3 (A) UV-vis absorption of BBR in PCPC/BBR at different time and (B) in vitro release profile of BBR from PCPC/BBR.

***Q4:*** *What dose of BBR adsorbed in the scaffolds is most helpful for promoting proliferation and differentiation of BMSCs? A BBR dose-dependent cell culture study is needed.*

**Answer:** Thanks for the reviewer’s precious comments. We have added some experimental data (Fig. S1) in there.

***Q5:*** *Figure 2. CLSM images: What’s the size of the scale bars?*

**Answer:** We greatly thank the reviewer for the precious comments which have contributed greatly to our revised manuscript. Due to the compression of the image, the scale is blurred. We have corrected this in the revised manuscript.

***Q6:*** *Figure 4-7, CLSM images: Missing scale bars*

**Answer:** We greatly thank the reviewer for the precious comments which have contributed greatly to our revised manuscript. Due to the compression of the image, the scale is blurred. We have corrected this in the revised manuscript.

***Q7:*** *Animal study: The authors need to provide the approval code for the experiments.*

**Answer:** Thank the reviewer very much for pointing out this. Ethical permission letter is listed in the following image.

***Q8:*** *8. Cite some relevant literature on the application of BBR delivery in bone and cartilage repair to highlight the importance of this study (https://doi.org/10.1016/j.matdes.2020.108540; https://doi.org/10.1016/j.carbpol.2018.11.050; https://doi.org/10.1007/s10439-016-1600-z).*

**Answer:** We greatly appreciate the reviewer’s precious comments. The suggested excellent papers about bone regeneration have been cited.

**Reviewer: 3**

***Q1:*** *In introduction section, Osteoporosis is a common systemic metabolic bone disease, usually causes bone loss, increased bone fragility and bone microstructure damage.*

**Answer:** We greatly appreciated the reviewer’s valuable suggestion. We have corrected this in the revised manuscript.

***Q2:*** *It is needed to accurately describe different words, such as porous hydroxyapatite ceramics scaffold (named PCPC), calcium phosphate cement (CPCs), porous calcium phosphate cement (PCPC). They are different materials.*

**Answer:** We greatly appreciated the reviewer’s valuable suggestion. We have corrected this in the revised manuscript.

***Q3:*** *Sprague–Dawley (SD) rats? Please provide full name.*

**Answer:** We greatly appreciated the reviewer’s valuable suggestion. We have corrected this in the revised manuscript.

***Q4:*** *It's best to prove whether the rats have osteoporosis in section 2.2 Animal experiments.*

**Answer:** We greatly appreciated the reviewer’s valuable suggestion. We have corrected this in the revised manuscript.

***Q5:*** *It is very confused from section 2.4 to section 2.8. The cell culturing times were different in different sections. It needs further confirmed.*

**Answer:** We greatly appreciated the reviewer’s valuable suggestion. We have corrected this in the revised manuscript.

***Q6:*** *In conclusion, “At the same time, PCPC can promote the expression of Runx2, BMP2, OCN and ALP.” PCPC should be PCPC / BBR? Because the authors are talk about PCPC / BBR.*

**Answer:** We greatly appreciated the reviewer’s valuable suggestion. We have corrected this in the revised manuscript.

***Q7:*** *In conclusion, more results need be included. It is too simple.*

**Answer:** .We greatly appreciated the reviewer’s valuable suggestion. We have corrected this in the revised manuscript.

***Q8:*** *For materials characterization, it is better to show the pore size, porosity of the scaffolds. And it is HA, β-TCP or BCP? Because calcium phosphate is the common name.*

**Answer:** We greatly appreciated the reviewer’s valuable suggestions. We obtained the the pore size, porosity of the scaffolds through the Brunner−Emmet−Teller (BET) measurements. The results showed that the pore size of PCPC was 20.4 nm and the porosity was 0.1164%, while the porosity of 0.6137% was achieved for PCPC/BBR with the pore size of 17.2 nm. We used XRD to test the diffraction peaks of the sample (Figure 1J). Compared with the standard card, the test result showed that the material used was biphasic calcium phosphate (BCP), which was composed of beta-tricalcium phosphate (β-TCP) and hydroxylapatite (HAP). (Standard spectral cards of β-TCP and HAP were PDF#09-0169 and PDF#72-1243 respectively).

***Q9:*** *Some close researches are suggested to be cited in this study.*

**Answer:** We greatly appreciated the reviewer’s valuable suggestion. We have corrected this in the revised manuscript.

**Review Editor**

Comments to the Author:

Based on the referees' reports and also my own reading, major revision is requiered before the publication. The author must address the deficiencies noted above. I also suggest the authors notice the relevant publications of the journal of Regenerative Biomaterials published during 2019-2021 in their revised manuscript.

**Answer:** We greatly appreciate the Editor’s precious comment. The relevant publications of the journal of Regenerative Biomaterials published during 2019-2021 have been cited in the revised manuscript.
